# Probiotic Supplementation With *Bifidobacterium longum* Subsp. *Longum* BL21 Improves Glycemic Control and Modulates Gut Microbiota in Type 2 Diabetes: A Randomized Controlled Trial

**DOI:** 10.1002/fsn3.71437

**Published:** 2026-01-09

**Authors:** Chengsheng Zhu, Yinhua Liu, Yanyan Chen, Zhiying Wang, Ya Gao, Fei Xu, Junyi Huang

**Affiliations:** ^1^ College of Food Science and Technology Henan University of Technology Zhengzhou China; ^2^ Department of Integrated Traditional Chinese and Western Medicine The First Affiliated Hospital of Zhengzhou University Zhengzhou China; ^3^ Food Nutrition and Chronic Disease, Intervention Laboratory, School of Life Sciences Shanghai University Shanghai China

**Keywords:** *Bifidobacterium longum* subsp. *longum*, glycemic control, gut microbiota modulation, type 2 diabetes mellitus

## Abstract

Type 2 diabetes mellitus (T2DM) remains a major global health challenge, with emerging evidence highlighting the therapeutic potential of gut microbiota modulation. This study aimed to evaluate the efficacy and safety of *Bifidobacterium longum* subsp. *longum* BL21 as an adjunctive therapy to metformin in patients with T2DM. In this 12‐week, randomized, double‐blind, placebo‐controlled trial, patients with T2DM received either metformin plus BL21 or metformin plus placebo. The primary endpoint was the change in hemoglobin A1c (HbA1c), while secondary endpoints included changes in fasting blood glucose (FBG), insulin resistance, lipid profiles, and gut microbiota composition. Supplementation with BL21 led to a significant reduction in HbA1c levels (*p* = 0.026) compared with placebo. Non‐significant trends toward reduction were also observed for FBG and insulin resistance. The intervention was well tolerated, with a safety profile comparable to placebo. Gut microbiota analysis revealed an increase in beneficial genera, including *Bifidobacterium* and *Faecalibacterium*, alongside a reduction in pathogenic taxa in the BL21 group. As an adjunct to metformin, BL21 supplementation improved glycemic control and modulated gut microbiota in patients with T2DM. These findings support the potential of BL21 as a novel adjunctive strategy for T2DM management, warranting further validation in larger and longer‐term studies.

**Clinical Trial Registration Number**: ChiCTR2300073299.

## Graphical abstract

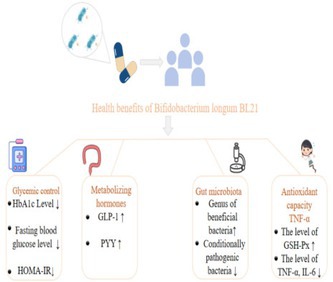

## 1 Introduction

Type 2 diabetes mellitus (T2DM) is a chronic metabolic disorder characterized by insulin resistance, β‐cell dysfunction, and impaired glucose metabolism (Diane et al. 2022; Liu et al. 2025). The global prevalence of T2DM has been rising steadily, establishing it as one of the most significant public health challenges worldwide. According to the International Diabetes Federation (IDF), approximately 537 million adults aged 20–79 years were living with diabetes in 2021, with T2DM accounting for over 90% of cases (Fernández‐Cao et al. 2024; Abd El‐Razik Siam et al. 2023). This number is projected to increase to 783 million by 2045, primarily driven by aging populations, sedentary lifestyles, and rising obesity rates, particularly in low‐ and middle‐income countries (Sun et al. 2022). Despite the availability of a wide range of pharmacological treatments, including metformin, sulfonylureas, and newer agents such as glucagon‐like peptide‐1 (GLP‐1) receptor agonists, a substantial proportion of patients fail to achieve optimal glycemic control, underscoring the need for adjunctive therapeutic strategies (Sundararaman et al. 2025; Olanrewaju et al. 2023).

Accumulating evidence indicates that the gut microbiota plays a critical role in the development and progression of T2DM (Tanase et al. 2020). Individuals with T2DM typically exhibit a disrupted gut microbial composition, characterized by a reduction in beneficial taxa (e.g., *Bifidobacterium*, *Akkermansia*) and an overgrowth of potentially pathogenic bacteria (e.g., *Enterobacteriaceae*) (Tanase et al. 2020). Such dysbiosis is believed to contribute to chronic systemic inflammation, impaired glucose metabolism, and insulin resistance (Belizário et al. 2018). Consequently, modulation of the gut microbiota has emerged as a promising therapeutic avenue to complement conventional diabetes treatments.

Among microbiota‐targeted interventions, probiotics have attracted considerable interest. Probiotics are defined as live microorganisms that confer health benefits to the host when administered in adequate amounts (Hill et al. 2014). Several clinical studies have investigated the impact of probiotic supplementation on metabolic outcomes in T2DM, with mixed results. While some studies demonstrated improvements in glycemic control, insulin sensitivity, and lipid profiles (Li et al. 2023; Ziegler et al. 2022; Wang et al. 2020), others reported no significant effects (Barengolts et al. 2019). These inconsistencies may be attributable to strain‐specific differences in probiotic efficacy and mechanisms of action.

*Bifidobacterium longum* subsp. *longum* BL21 has shown promising results in preclinical studies, demonstrating the ability to modulate gut microbiota composition, enhance intestinal barrier integrity, regulate glucose metabolism, and attenuate inflammation and oxidative stress (Gai et al. 2023; Hao et al. 2022). These findings suggest that BL21 may have potential clinical utility as an adjunctive therapy for T2DM. The present randomized, double‐blind, placebo‐controlled trial aimed to evaluate the efficacy and safety of BL21 supplementation as an adjunct to metformin therapy in patients with T2DM. The primary objective was to assess changes in hemoglobin A1c (HbA1c) levels, while secondary objectives included the evaluation of fasting blood glucose (FBG), insulin resistance, lipid profiles, and gut microbiota composition. We hypothesized that BL21 supplementation would enhance glycemic control and favorably modulate gut microbiota in patients with T2DM, thereby supporting its potential role in microbiota‐targeted metabolic therapies.

## 2 Materials and Methods

### 2.1 Study Design and Ethics

This was a multicenter, parallel‐group, randomized, double‐blind, placebo‐controlled trial conducted to evaluate the efficacy and safety of *Bifidobacterium longum* subsp. *longum* BL21 as an adjunctive therapy in patients with T2DM. The study was conducted in accordance with the Declaration of Helsinki and Good Clinical Practice guidelines and was approved by the Ethics Committee of the First Affiliated Hospital of Harbin Medical University (Approval No. IRB‐AF/SC‐04/02.0). The trial was registered with the Chinese Clinical Trial Registry (ChiCTR2300073299). Written informed consent was obtained from all participants prior to enrollment.

### 2.2 Participants and Recruitment

Eligible participants were adults aged 25–65 years with a body mass index (BMI) between 19.0 and 35.0 kg/m^2^, diagnosed with T2DM according to the 2019 World Health Organization (WHO) criteria (Artasensi et al. 2020). Participants were required to have stable glycemic control through diet, exercise, or stable doses of sulfonylureas or insulin for at least 3 months before enrollment. Inclusion criteria included glycated hemoglobin (HbA1c) levels between 6.5% and 10.0% and fasting plasma glucose (FPG) levels between 7.0 and 13.3 mmol/L.

Exclusion criteria included type 1 diabetes mellitus, active smoking, alcohol use disorder, autoimmune diseases, recent use (within 6 weeks) of antimicrobial agents, probiotics, proton pump inhibitors, or gastrointestinal motility modulators. Other exclusions included uncontrolled hypertension, coronary artery disease, chronic hepatic or renal disease, malignancy, active infection, participation in another clinical trial within the previous 3 months, pregnancy or lactation, and clinically significant abnormalities detected during screening assessments. Participants likely to be non‐compliant or exposed to similar functional interventions were also excluded.

### 2.3 Sample Size

The sample size was empirically estimated based on the design of a comparable 12‐week probiotic trial in T2D by Perraudeau et al. (Perraudeau et al. 2020), which demonstrated significant glycemic control with 76 participants. We enrolled 80 cases to approximate this benchmark while allowing for modest attrition.

### 2.4 Randomization and Blinding

Participants were randomized in a 1:1 ratio using a computer‐generated randomization sequence, stratified by baseline HbA1c levels and age. Both participants and investigators were blinded to group assignment. Identical capsules, indistinguishable in appearance, taste, and packaging, were used for the BL21 and placebo groups to maintain blinding throughout the study.

### 2.5 Intervention

All participants continued their baseline metformin therapy with dosage adjustments as clinically indicated. In addition to standard care, participants in the BL21 group received a daily oral supplement of BL21 (20 billion CFU per sachet, a dose anchored at the mid‐range of the 10^9^–10^11^ CFU/day efficacy window identified in a 2021 systematic review (Zhang et al. 2022), corroborated by two independent studies (Zhao et al. 2025; Geng et al. 2025) that reported significant glycemic and lipid benefits using the same dose), while those in the placebo group received identical‐appearing preparation containing inert ingredients. The intervention period lasted 12 weeks. Participants were instructed to maintain their usual diet and physical activity levels during the study. The probiotic and placebo samples were provided by Wecare Probiotics Co. Ltd.

### 2.6 Outcome Measures

#### 2.6.1 Primary Outcome

The primary outcome was the change in HbA1c levels from baseline to week 12, measured using High Performance Liquid Chromatography (HPLC) at a central certified laboratory (Bergmann and Sypniewska 2016).

#### 2.6.2 Secondary Outcomes

Secondary outcomes included changes in glycemic control parameters (fasting blood glucose [FBG], insulin resistance assessed by the Homeostasis Model Assessment of Insulin Resistance [HOMA‐IR], and fasting insulin levels [FINS]), metabolic profiles (total cholesterol [TC], triglycerides [TG], high‐density lipoprotein cholesterol [HDL‐C], low‐density lipoprotein cholesterol [LDL‐C], and high‐sensitivity C‐reactive protein [hs‐CRP]), and safety indicators. Safety assessments encompassed monitoring of adverse events (AEs) and evaluation of laboratory parameters, including hematologic markers (white blood cell count, red blood cell count, and platelet count), liver function tests (alanine aminotransferase [ALT] and aspartate aminotransferase [AST]), renal function markers (serum creatinine and urea), electrolyte levels (sodium, potassium, and chloride), and urinalysis results (proteinuria, hematuria, leukocyturia, and ketonuria).

### 2.7 Gut Microbiota Analysis

Stool samples were collected at baseline and at week 12. Genomic DNA was extracted using the QIAamp Fast DNA Stool Mini Kit (QIAGEN, Hilden, Germany) following the manufacturer's protocol (Kaisar et al. 2017). The hypervariable V3–V4 regions of the bacterial 16S rRNA gene were amplified by polymerase chain reaction (PCR) and sequenced using the Illumina MiSeq platform (Dong et al. 2023). Alpha diversity indices (Chao1, Ace, Shannon, Simpson) were calculated to assess microbial richness and evenness. Beta diversity was evaluated using Principal Coordinate Analysis (PCoA) based on Bray–Curtis dissimilarity matrices (Oksanen 2010). Mantel tests were performed to analyze the correlation between microbial community changes and clinical outcomes.

### 2.8 Statistical Analysis

All statistical analyses were conducted using R software (version 4.3.2) (Team RDC 2010). The Shapiro–Wilk test was used to assess the normality of continuous variables. Normally distributed continuous data were presented as mean ± standard deviation (SD), and comparisons were made using independent *t*‐tests. Non‐normally distributed continuous data were presented as median (interquartile range, IQR), with comparisons conducted using the Mann–Whitney *U* test. Paired *t*‐tests or Wilcoxon signed‐rank tests were used to assess within‐group changes from baseline. Categorical variables were summarized as counts (percentages) and compared using the chi‐square test or Fisher's exact test as appropriate. For gut microbiota analyses, independent *t*‐tests or Kruskal–Wallis tests were used to compare diversity indices between groups. A two‐sided *p*‐value < 0.05 was considered statistically significant.

## 3 Results

### 3.1 Baseline Characteristics

**FIGURE 1:**
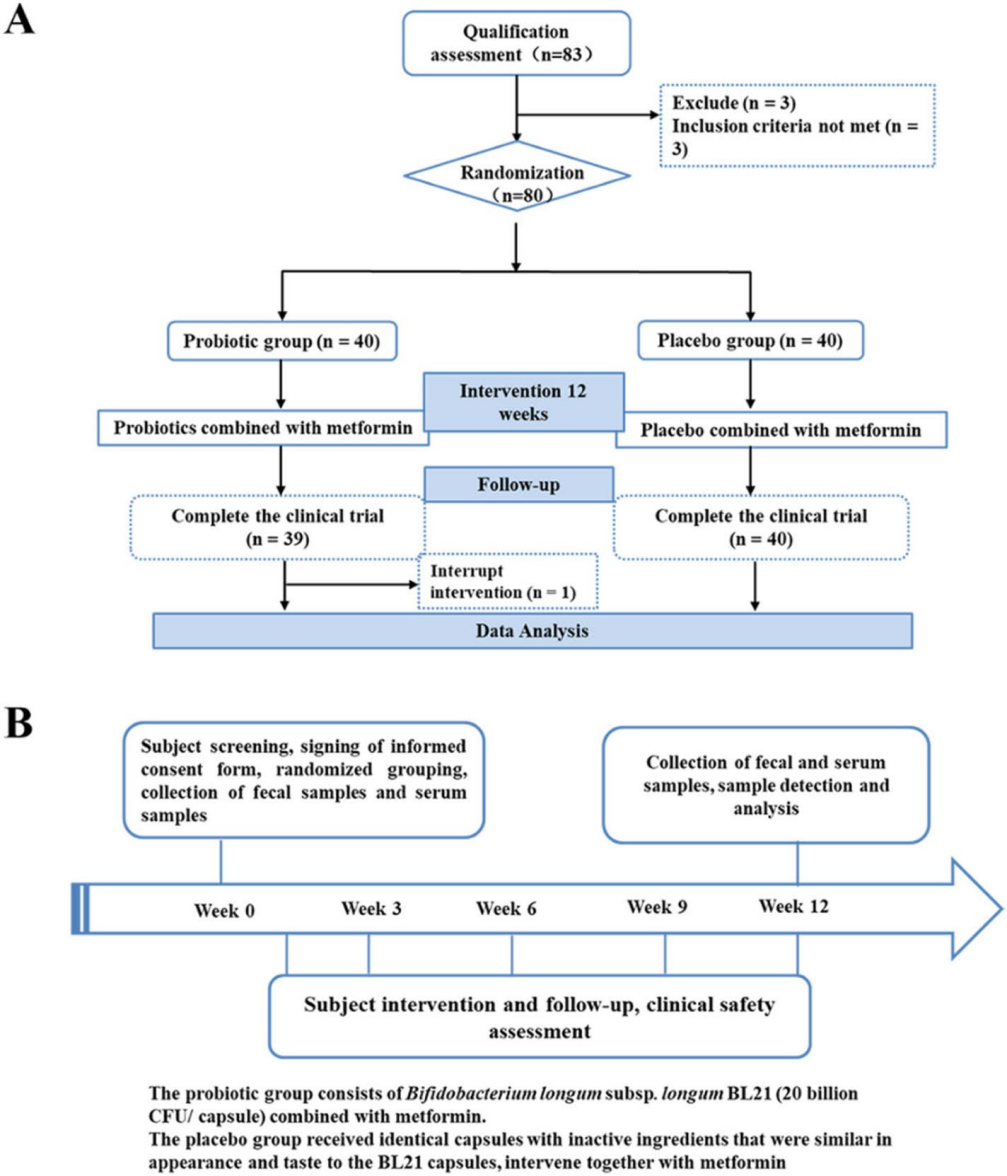
Flowchart for this study. Process of the study implementation. (A) The design of the study. (B) Flow diagram of this study selection.

At the beginning of the study, a total of 83 individuals were screened, of whom 80 met the eligibility criteria and were randomized into two groups: BL21 group (*n* = 40) and placebo group (*n* = 40). During the intervention period, one participant from the BL21 group withdrew due to personal reasons, resulting in 79 participants completing the 12‐week study (Figure 1). Table 1 summarizes the baseline demographic and clinical characteristics of the participants. There were no significant differences between the BL21 and placebo groups in gender distribution, age, body mass index (BMI), or the prevalence of comorbidities, including coronary heart disease (CHD) and hypertension (BP) (all *p* > 0.05). Specifically, the proportion of women was 40.0% in the placebo group and 48.7% in the BL21 group (*p* = 0.580). The mean age was 56.7 years in the placebo group and 59.2 years in the BL21 group (*p* = 0.341). The mean BMI was 25.7 and 25.1 in the placebo and BL21 groups, respectively (*p* = 0.331). No participants reported alcohol consumption. These findings confirm that randomization was successful and that the groups were comparable at baseline.

**TABLE 1:** Baseline characteristics of patients with T2DM at enrollment.

	Placebo (*n* = 40)	BL21 (*n* = 39)	*p*
Gender, *n* (%)			
Female	16 (40.0%)	19 (48.7%)	0.580
Male	24 (60.0%)	20 (51.3%)	
Age, years, mean (SD)	56.7 (10.2)	59.2 (13.0)	0.341
BMI, kg/m^2^, mean (SD)	25.7 (2.6)	25.1 (2.7)	0.331
Smoking, *n* (%)	9 (22.5%)	7 (17.9%)	0.823
Alcohol consumption			
No *n* (%)	40 (100.0%)	39 (100.0%)	—
CHD, *n* (%)	5 (12.5%)	3 (7.7%)	0.712
BP, *n* (%)	16 (40.0%)	18 (46.2%)	0.745

*Note:* *Bifidobacterium longum* subsp. *longum* BL21, BL21. Values are presented as mean (standard deviation, SD) for continuous variables and number (percentage) for categorical variables. *p*‐values were calculated using independent *t*‐tests for continuous variables and chi‐square tests for categorical variables.

Abbreviations: BP, blood pressure (hypertension); CHD, coronary heart disease.

### 3.2 Safety Profile

The safety profile of BL21 supplementation was comparable to that of metformin monotherapy. As shown in Table 2, there were no significant differences between groups in hematologic parameters, liver enzyme levels (ALT, AST), renal function markers (urea, creatinine), or electrolyte levels (sodium, potassium, chloride) (all *p* > 0.05). Urinalysis revealed a higher proportion of negative findings for blood (BLD) and leukocytes (LEU) in the placebo group at both baseline and post‐intervention (*p* < 0.01), although these differences were not considered clinically significant. Additionally, transient ketonuria (KET) was observed in 5.6% of participants in the BL21 group compared to 7.5% in the placebo group (*p* = 0.325), with no significant differences between groups.

**TABLE 2:** Comparison of safety parameters in BL21 and Placebo groups.

	Placebo group (*n* = 40)		BL21 group (*n* = 39)		*p*
	Baseline	End	Baseline	End	
WBC	6.4 (1.4)	6.3 (1.2)	6.2 (1.5)	6.2 (1.6)	0.970
NEUT	3.6 (1.0)	3.5 (0.9)	3.6 (1.0)	3.6 (1.1)	0.955
LYMPH	2.1 (0.6)	2.2 (0.6)	2.0 (0.6)	2.0 (0.5)	0.334
MONO	0.4 (0.1)	0.4 (0.1)	0.5 (0.1)	0.4 (0.1)	0.699
E0	0.2 (0.1)	0.1 (0.1)	0.2 (0.2)	0.2 (0.2)	0.789
BASO	< 0.1 (< 0.1)	< 0.1 (< 0.1)	< 0.1 (< 0.1)	< 0.1 (< 0.1)	0.973
RBC	5.0 (0.4)	4.9 (0.9)	4.8 (0.5)	4.9 (0.5)	0.659
HGB	148.0 (17.6)	149.0 (18.3)	147.7 (14.7)	149.1 (15.6)	0.977
PLT	242.9 (74.3)	242.5 (73.7)	231.0 (63.8)	238.7 (66.4)	0.864
ALT	26.3 (14.7)	27.4 (15.4)	25.5 (18.5)	23.7 (11.4)	0.745
AST	24.0 (6.5)	25.1 (7.2)	25.4 (12.9)	23.8 (7.6)	0.822
ALB	44.6 (2.8)	44.3 (4.7)	42.8 (3.3)	43.6 (3.5)	0.119
TBIL	15.1 (5.8)	15.2 (5.5)	15.5 (5.1)	14.4 (4.7)	0.853
DBIL	2.5 (1.0)	2.6 (1.0)	2.7 (0.8)	2.8 (1.1)	0.718
IBIL	12.6 (4.9)	12.6 (4.7)	12.8 (3.7)	12.2 (4.0)	0.953
UREA	5.9 (1.5)	6.0 (1.6)	5.7 (1.8)	5.9 (1.5)	0.868
Cr	68.0 (18.2)	68.6 (19.3)	68.8 (18.0)	67.9 (21.2)	0.995
UA	354.8 (113.8)	346.1 (84.3)	347.9 (116.1)	344.9 (114.9)	0.977
Na	139.3 (2.0)	139.6 (1.6)	139.6 (2.1)	140.2 (1.6)	0.193
K	4.3 (0.3)	4.2 (0.3)	4.2 (0.4)	4.3 (0.4)	0.494
Cl	103.2 (2.4)	104.3 (6.4)	104.1 (1.9)	104.6 (1.8)	0.346
SG	1.0 (< 0.1)	1.0 (< 0.1)	1.0 (< 0.1)	1.0 (< 0.1)	0.594
PRO: Negative	31 (77.5%)	34 (85.0%)	27 (69.2%)	27 (75.0%)	0.417
BLD: Negative	38 (95.0%)	39 (97.5%)	27 (69.2%)	26 (72.2%)	< 0.001
LEU: Negative	37 (92.5%)	37 (92.5%)	27 (69.2%)	26 (72.2%)	0.006
OB: Negative	37 (92.5%)	35 (87.5%)	33 (84.6%)	32 (88.9%)	0.745
KET: Negative	39 (97.5%)	37 (92.5%)	39 (100.0%)	34 (94.4%)	0.325
GLU: Negative	18 (45.0%)	15 (37.5%)	23 (59.0%)	18 (50.0%)	0.278

*Note:* *Bifidobacterium longum* subsp. *longum* BL21, BL21. Data presented as mean (SD) for continuous variables or *n* (%) for categorical variables. *p*‐values reflect interaction effects between group and time from mixed‐effects models (continuous) or generalized estimating equations (categorical).

Abbreviations: ALB, albumin; ALT, alanine aminotransferase; AST, aspartate aminotransferase; Cr, creatinine; TBIL, total bilirubin.

### 3.3 Glycemic Control and Metabolic Parameters

As presented in Tables 3 and 4, the BL21 group exhibited greater improvement in glycemic control than the placebo group. The mean HbA1c level decreased from 7.6% (SD = 1.6) at baseline to 7.1% (SD = 1.2) at week 12 in the BL21 group, whereas only a minimal reduction was observed in the placebo group (7.5% [SD = 1.3] to 7.4% [SD = 1.3]). The difference in HbA1c change between groups was statistically significant (*p* = 0.026), with a median reduction of −0.3% in the BL21 group versus 0.0% (IQR: −0.4% to 0.3%) in the placebo group.

**TABLE 3:** Glycemic control and metabolic parameters in the BL21 and Placebo groups.

	Placebo group (*n* = 40)		BL21 group (*n* = 39)		*p*
	Baseline	End	Baseline	End	
HbA1c	7.5 (1.3)	7.4 (1.3)	7.6 (1.6)	7.1 (1.2)	0.415
FBG	8.6 (2.6)	7.9 (2.3)	8.8 (2.7)	7.7 (1.5)	0.126
HOMAIR	8.3 (12.3)	8.6 (15.4)	8.4 (12.6)	7.1 (9.8)	0.959
FINS	21.2 (29.7)	22.0 (32.8)	18.9 (21.6)	19.4 (23.8)	0.955
Cpep	2.7 (1.9)	2.4 (1.6)	2.9 (1.3)	2.6 (1.2)	0.472
CRP	3.2 (1.7)	3.2 (2.0)	3.6 (2.6)	3.0 (1.8)	0.714
TC	4.8 (1.2)	4.6 (1.1)	5.0 (1.2)	4.9 (1.1)	0.6
TG	1.9 (1.2)	2.1 (1.3)	1.9 (1.2)	1.8 (1.1)	0.627
HDL‐C	1.3 (0.3)	1.3 (0.3)	1.3 (0.3)	1.3 (0.3)	0.924
LDL‐C	3.0 (0.9)	2.9 (0.9)	3.1 (0.9)	3.2 (1.0)	0.585

*Note:* *Bifidobacterium longum* subsp. *longum* BL21, BL21. Data presented as mean (SD).

Abbreviations: CRP, C‐reactive protein; FBG, fasting blood glucose; HbA1c, glycated hemoglobin; HOMA‐IR, homeostatic model for assessing insulin resistance; TG, triglycerides.

**TABLE 4:** Differences between the groups in the changes in metabolic parameters from baseline to week 12.

	Placebo group (*n* = 40)	BL21 group (*n* = 39)	*p*
HbA1c	0.0 [−0.4–0.3]	−0.3 [−0.8–0.0]	0.026
FBG	−0.4 [−1.1–0.3]	−0.9 [−2.0–0.4]	0.305
HOMAIR	−0.3 [−1.7–0.6]	−0.6 [−1.6–0.4]	0.456
FINS	0.2 [−2.0–2.1]	−0.2 [−1.7–1.1]	0.342
Cpep	−0.3 [−0.6–0.1]	−0.3 [−0.7–0.2]	0.713
CRP	−0.2 [−1.0–0.7]	−0.1 [−1.0–0.2]	0.524

*Note:* *Bifidobacterium longum* subsp. *longum* BL21, BL21. Data presented as median [IQR] for non‐normally distributed variables; *p*‐values derived from Mann–Whitney *U*‐tests.

Abbreviations: FBG, fasting blood glucose; HbA1c, glycated hemoglobin; HOMA‐IR, homeostatic model for the assessment of insulin resistance.

The BL21 group also showed a greater reduction in fasting blood glucose (FBG) levels (Δ‐1.1 mmol/L) compared to the placebo group (Δ‐0.7 mmol/L), although this difference did not reach statistical significance (*p* = 0.126). Similarly, insulin resistance as assessed by HOMA‐IR decreased by 1.3 units in the BL21 group but slightly increased in the placebo group; however, this difference was not statistically significant (*p* = 0.959).

In terms of lipid profiles, TG levels decreased slightly in the BL21 group (Δ‐0.1 mmol/L) but increased in the placebo group (Δ + 0.2 mmol/L), with no significant difference between groups (*p* = 0.627). Hs‐CRP levels decreased by 16.7% in the BL21 group, whereas no substantial change was observed in the placebo group, although the between‐group difference was not statistically significant (*p* = 0.714).

### 3.4 Gut Microbiota Analysis

**FIGURE 2:**
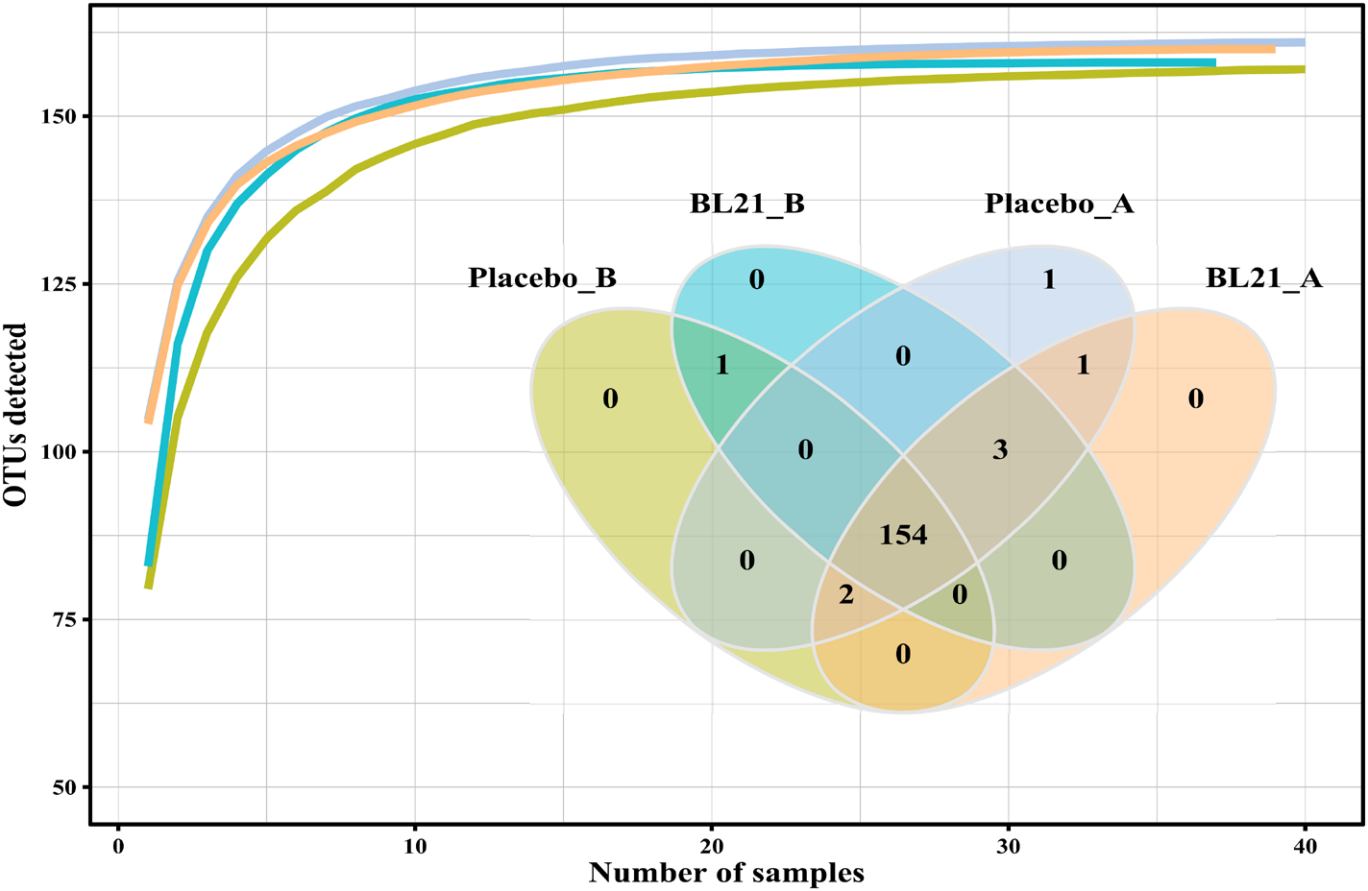
Analysis of species accumulation and shared species. Species accumulation curves and Venn diagram showing the shared gut microbial species between the placebo and BL21 groups. *Bifidobacterium longum* subsp. *longum* BL21, BL21.

**FIGURE 3:**
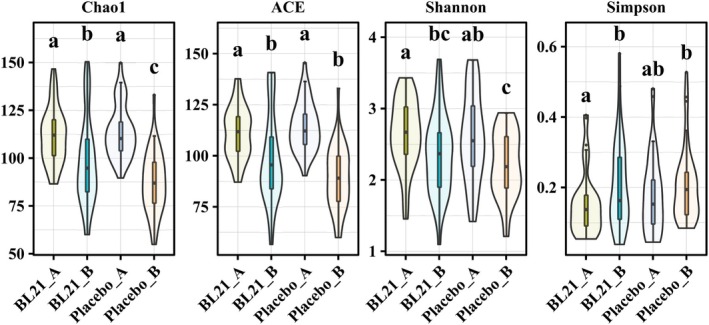
Alpha diversity analysis of the gut microbiota. Alpha diversity metrics (Chao1, Ace, Shannon, and Simpson indices) comparing the richness and diversity of the gut microbiota between the placebo and BL21 groups at baseline and post‐intervention. *Bifidobacterium longum* subsp. *longum* BL21, BL21.

**FIGURE 4:**
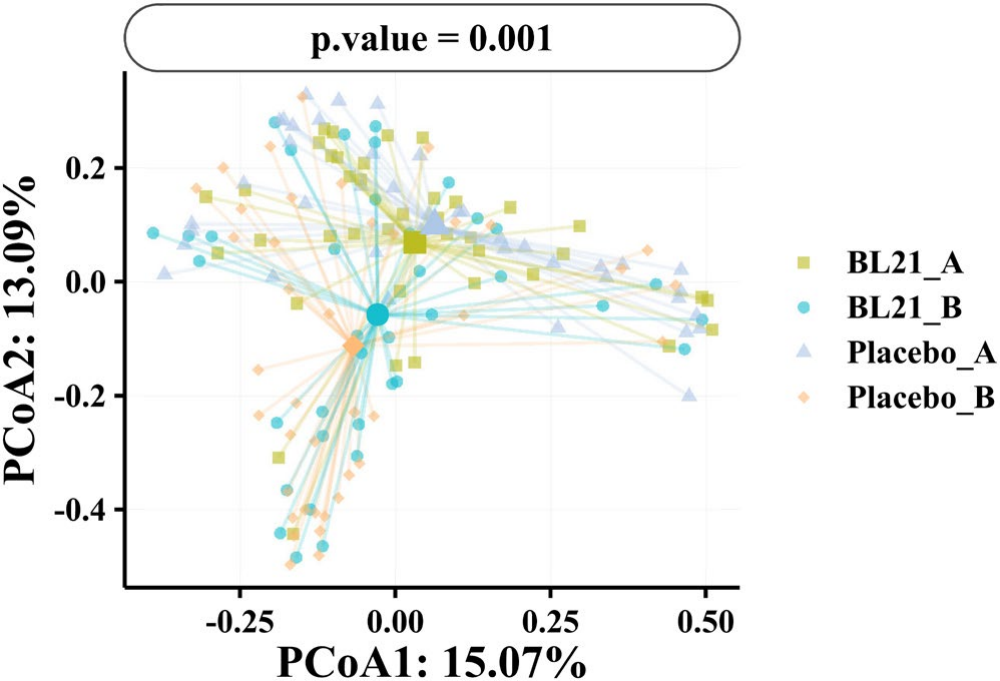
Beta‐diversity analysis of the composition of the gut microbiota. Principal coordinate analysis (PCoA) shows the differences in gut microbiota composition between the placebo and BL21 groups at different time points. *Bifidobacterium longum* subsp. *longum* BL21, BL21.

Analysis of gut microbiota composition indicated beneficial effects of BL21 supplementation on microbial diversity and structure. At baseline, no significant differences were observed between groups in species richness and diversity indices (Figure 2). Following 12 weeks of intervention, the BL21 group exhibited significantly higher alpha diversity, as reflected by the Chao1 index (*p* < 0.05), compared to the placebo group (Figure 3). Although trends toward higher diversity were observed in the BL21 group based on the Shannon and Simpson indices, these differences were not statistically significant. Beta diversity analysis (Figure 4) revealed distinct clustering patterns between the groups at the midpoint of the study; however, no significant differences were detected at the end of the intervention period (*p* = 0.253).

**FIGURE 5:**
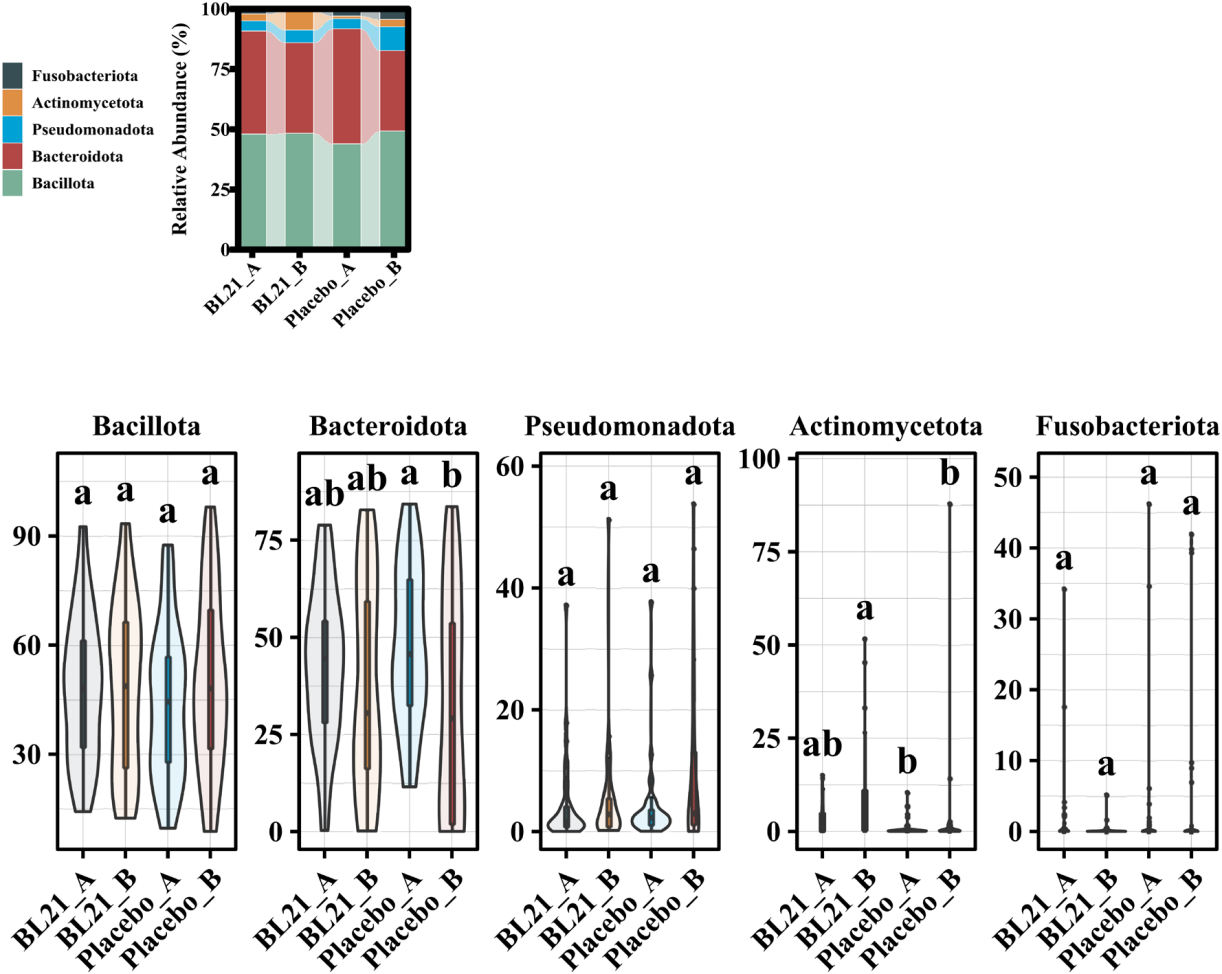
Analysis of the intestinal microbiota at phylum level. Relative abundance of the main bacterial phyla in the gut microbiota of participants in the placebo and BL21 groups at baseline and post‐intervention. *Bifidobacterium longum* subsp. *longum* BL21, BL21.

At the phylum level, the relative abundance of Bacteroidota decreased significantly in the placebo group, whereas the BL21 group maintained a more stable phylum profile (Figure 5). In addition, Actinomycetota abundance was significantly higher in the placebo group after the intervention (*p* < 0.05).

At the genus level, BL21 supplementation preserved the relative abundance of beneficial genera such as *Faecalibacterium*, while the placebo group exhibited a significant reduction. The relative abundance of *Bifidobacterium* also increased significantly in the BL21 group. Conversely, potentially pathogenic genera, including *Veillonella*, *Clostridium*, and *Escherichia/Shigella*, decreased following BL21 supplementation, whereas no significant changes were observed in the placebo group (Table S1).

**FIGURE 6:**
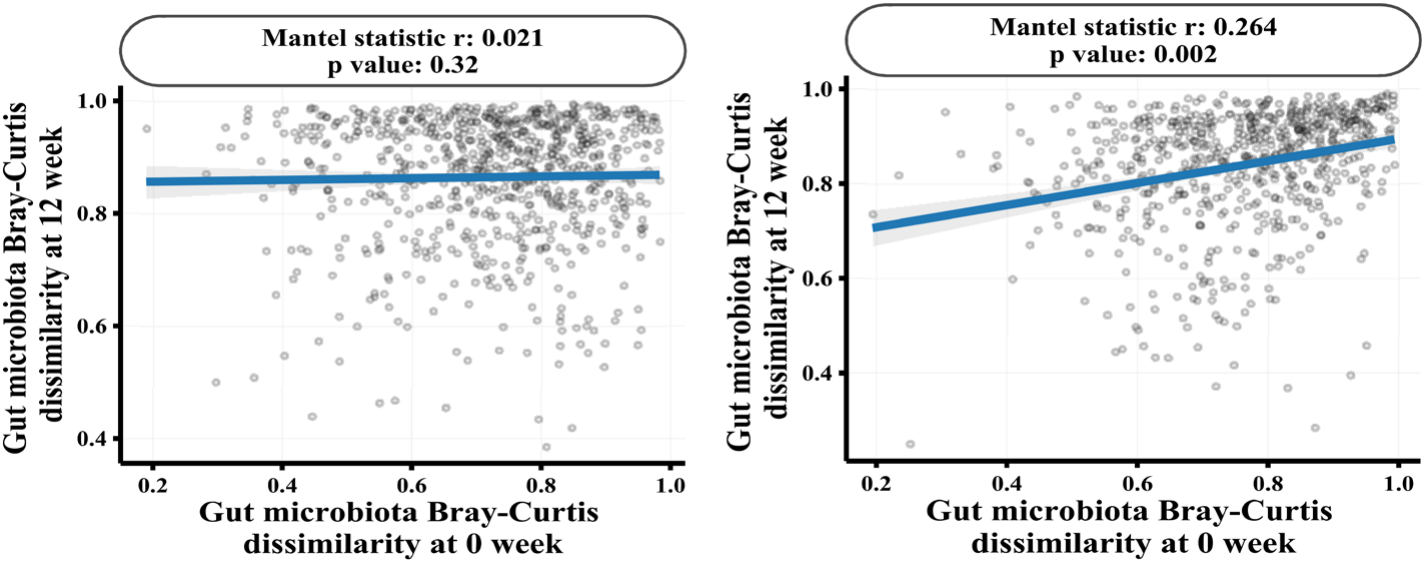
Mantle correlation analysis of the stability of the gut microbiota. Mantel correlation analysis to evaluate the correlation of the abundance of the intestinal microbiota before and after treatment in the placebo (A) and BL21 (B) groups. *Bifidobacterium longum* subsp. *longum* BL21, BL21.

Mantel correlation analysis (Figure 6) demonstrated a significant correlation between baseline and post‐treatment microbial compositions in the BL21 group (*p* = 0.002, *r* = 0.264), suggesting that BL21 supplementation contributed to the stabilization of gut microbiota structure. This effect was not observed in the placebo group (*p* = 0.32).

## 4 Discussion

This randomized, double‐blind, placebo‐controlled trial demonstrated that supplementation with BL21 as an adjunct to metformin significantly improved glycemic control in patients with T2DM. Specifically, BL21 supplementation led to a clinically meaningful reduction in HbA1c by 0.3% compared with metformin monotherapy, aligning with the American Diabetes Association's minimum clinically important difference (MCID) threshold of 0.3%–0.5% for therapeutic benefit (Dankers et al. 2021). However, it is important to critically evaluate this finding in the context of the non‐significant changes in secondary glycemic and metabolic parameters, such as FBG, HOMA‐IR, and TG. The isolated reduction in HbA1c, while statistically significant and meeting the MCID threshold, suggests a modest but relevant glycemic benefit. When compared to established adjunctive therapies for T2DM, such as some newer antihyperglycemic agents which often demonstrate HbA1c reductions of 0.5%–1.0% or more, the effect size observed with BL21 is smaller. Nevertheless, even modest HbA1c reductions are associated with decreased long‐term microvascular complications, and the potential for a probiotic intervention to confer this benefit with a favorable safety profile represents a valuable non‐pharmacological adjunct. Future studies should aim to identify patient subgroups that may derive greater benefit from BL21 supplementation and explore whether longer intervention durations or synergistic combinations with other therapies could amplify these effects. Although improvements in FBG, HOMA‐IR, and TG levels were observed in the BL21 group, these changes did not reach statistical significance. In addition to glycemic improvements, BL21 supplementation favorably modulated gut microbiota composition and was well tolerated, with a safety profile comparable to that of metformin alone.

Our findings contribute to the growing body of evidence from randomized controlled trials (RCTs) investigating probiotics for T2DM management (Li et al. 2023; Dong et al. 2023; Oksanen 2010). The observed HbA1c reduction of 0.3% with BL21 supplementation is consistent with the effects reported in several meta‐analyses, which typically show HbA1c reductions in the range of 0.2%–0.6% for various probiotic interventions (Li et al. 2023; Dankers et al. 2021). However, a critical distinction of our study lies in its evaluation of a single‐strain probiotic. Many prior RCTs demonstrating significant benefits utilized multi‐strain formulations, often combining various Lactobacillus and Bifidobacterium species (Team RDC 2010; Dankers et al. 2021). While such combinations may theoretically target multiple mechanisms simultaneously, they complicate the attribution of efficacy to any specific strain and pose challenges for standardizing mechanisms of action and consistent manufacturing. In this context, the significant glycemic improvement achieved with *Bifidobacterium longum* subsp. longum BL21 alone underscores its inherent potency and suggests that a targeted, single‐strain approach can be effective. This enhances its appeal as a well‐defined therapeutic candidate, as the observed effects can be unequivocally linked to this specific strain, facilitating a clearer understanding of its mechanism of action and a more straightforward path for product standardization and regulatory approval. In addition to glycemic improvements, BL21 supplementation led to favorable shifts in gut microbiota composition, aligning with previous observations that probiotics can restore microbial balance in metabolic disorders (Dong et al. 2023, 2024). Specifically, we observed significant increases in the relative abundance of *Bifidobacterium* and *Faecalibacterium* genera known to be associated with enhanced insulin sensitivity and reduced systemic inflammation (Ikeda et al. 2022; Ragavan and Hemalatha 2024). Concurrently, there was a decrease in potentially pathogenic genera such as *Escherichia/Shigella* and *Veillonella*, which have been implicated in chronic low‐grade inflammation and insulin resistance (Pham et al. 2022; Du et al. 2024). These microbial changes are consistent with the hypothesis that probiotic‐mediated modulation of gut microbiota may contribute to improved glucose metabolism, partly through the enhancement of beneficial bacterial populations and suppression of pro‐inflammatory taxa (Cristofori et al. 2021; Wang et al. 2015).

One plausible mechanism underlying the metabolic benefits is the modulation of gut microbiota composition and the consequent enhancement of short‐chain fatty acid (SCFA) production (Morrison and Preston 2016; Akhtar et al. 2022). SCFAs, particularly butyrate, are primarily synthesized by beneficial gut bacteria such as *Bifidobacterium* and *Faecalibacterium* (Ikeda et al. 2022; Ragavan and Hemalatha 2024), both of which were significantly enriched following BL21 supplementation in this study. This is consistent with findings from other probiotic strains, such as *Lactiplantibacillus plantarum* T34, which has been shown to enhance intestinal barrier function and restore gut homeostasis, further supporting the role of specific probiotics in maintaining gut integrity and metabolic health (Hao et al. 2025). SCFAs have been shown to exert multiple beneficial effects relevant to glucose metabolism, including improving insulin sensitivity (Pham et al. 2022), reducing systemic inflammation (Du et al. 2024), and maintaining the integrity of the intestinal barrier (Liu et al. 2021). Through these mechanisms, SCFAs contribute to the prevention of insulin resistance and the stabilization of metabolic homeostasis (Portincasa et al. 2022). Moreover, the significant increase in microbial richness observed in the BL21 group, as indicated by the Chao1 index, and the stabilization of gut microbiota composition over time further support the notion that BL21 promotes a healthier and more resilient gut ecosystem. Notably, the reduction in pathogenic genera such as Escherichia/Shigella observed in our study may also be linked to antimicrobial mechanisms similar to those reported for encapsulated bioactive compounds, such as caffeic acid phenethyl ester delivered via lipid nanocapsules, which exhibit enhanced antibacterial efficacy through controlled release (Hao et al. 2025). By fostering a favorable microbial environment rich in SCFA‐producing taxa and reducing the prevalence of pro‐inflammatory genera, BL21 may indirectly enhance gut barrier function, attenuate chronic low‐grade inflammation, and improve insulin signaling pathways, ultimately contributing to better glycemic control in patients with T2DM. It is important to note that the proposed role of SCFAs in mediating the effects of BL21, while supported by the observed taxonomic changes and the established literature, remains hypothetical in the context of this study, as we did not directly quantify SCFA levels or perform functional metagenomic analyses to validate this pathway.

BL21 supplementation was well tolerated, with no serious adverse events reported during the 12‐week intervention. The safety profile of BL21 was comparable to that of metformin monotherapy, with no significant differences observed in hematologic, hepatic, renal, or electrolyte parameters between groups. These findings are consistent with previous probiotic studies, which have generally demonstrated a favorable safety profile. The absence of clinically relevant adverse effects suggests that BL21 can be safely administered as a long‐term adjunctive therapy for T2DM, which is particularly important given the chronic nature of the disease and the need for sustained treatment strategies.

This study has several strengths. To our knowledge, it represents the first randomized, double‐blind, placebo‐controlled clinical trial to evaluate the efficacy and safety of BL21 as an adjunct to metformin in patients with T2DM. These results are consistent with previous studies demonstrating the favorable safety profiles of probiotic interventions in metabolic disorders.

The study design incorporated rigorous randomization and blinding procedures, and the comprehensive evaluation included both clinical metabolic endpoints and gut microbiota profiling, providing mechanistic insights into the observed effects. However, several limitations should be noted. First, the analysis of the gut microbiota was primarily taxonomic and descriptive. The lack of functional data, such as direct measurement of short‐chain fatty acid levels or metagenomic sequencing to infer microbial metabolic pathways (e.g., via PICRUSt or KEGG), limits our ability to draw definitive conclusions about the mechanistic links between BL21 supplementation and the observed metabolic benefits. Second, the relatively small sample size and the absence of a formal a priori power calculation constitute a methodological limitation. While our sample size was empirically estimated based on a comparable trial, the lack of prospective power analysis may affect the precision of our effect estimates and limit generalizability. Future confirmatory trials should incorporate formal sample size determination to ensure adequate statistical power. Third, the 12‐week intervention period may not be sufficient to fully assess the long‐term efficacy and sustainability of BL21‐induced improvements in glycemic control and gut microbiota composition. Although we observed significant improvements in HbA1c and favorable shifts in gut microbiota composition within this timeframe, it remains unclear whether these effects are maintained or enhanced over a longer duration. A longer follow‐up period would help elucidate the durability of glycemic control, the stability of microbial community structure, and the potential for further metabolic improvements, such as in insulin sensitivity and lipid metabolism, which may require more time to manifest fully. Fourth, this study focused on a single probiotic strain; given the strain‐specific nature of probiotic effects, further studies are needed to compare BL21 with other probiotic strains and combinations. Future research should aim to validate these findings in larger, multi‐center trials with longer follow‐up durations to better assess the long‐term efficacy and safety of BL21 supplementation in T2DM management. Comparative studies evaluating the effects of BL21 versus other probiotic strains or multi‐strain formulations would provide valuable insights into its relative efficacy. Additionally, mechanistic studies exploring the molecular pathways through which BL21 modulates gut microbiota, inflammation, and glucose metabolism could further elucidate its therapeutic potential and inform the development of microbiota‐targeted interventions for metabolic diseases.

Furthermore, to maximize the clinical efficacy of probiotic strains like BL21, future formulations could benefit from advanced encapsulation technologies. These strategies, as reviewed in the context of colorectal cancer (Yang et al. 2025), can protect probiotics from gastric acid and bile salts, enhance their viability, and promote targeted release in the colon, potentially leading to more potent and reproducible therapeutic outcomes.

## 5 Conclusion

In conclusion, this randomized controlled trial demonstrated that *Bifidobacterium longum* subsp. *longum* BL21 significantly improved glycemic control and modulated gut microbiota in patients with T2DM. The observed reduction in HbA1c, along with favorable trends in insulin resistance and lipid metabolism, suggests BL21 is a promising adjunct to conventional pharmacological treatments like metformin. By enhancing the abundance of beneficial gut microbiota and potentially reducing systemic inflammation, BL21 may contribute to improved metabolic health outcomes in T2DM. Given its favorable safety profile and therapeutic potential, further large‐scale, long‐term studies are warranted to confirm these findings and explore the broader clinical applications of BL21 in the management of metabolic diseases.

## Author Contributions

**Chengsheng Zhu:** conceptualization, investigation, formal analysis, fundraising. **Yinhua Liu** and **Zhiying Wang:** conceptualization, data maintenance, methodology, software, writing – original draft, writing – review and editing, acquisition of funds. **Yanyan Chen:** project management, software, writing – original design, resources, supervision, formal analysis, software, validation, visualization. **Ya Gao:** data curation, investigation. **Junyi Huang:** conceptualization, investigation, funding acquisition, writing – original draft, validation, visualization, methodology, resources. **Fei Xu:** conceptualization, writing – original draft, writing – review and editing, funding acquisition, investigation, methodology, validation, visualization, formal analysis, project administration, software, data curation, supervision, resources.

## Conflicts of Interest

The authors declare no conflicts of interest.

## Supporting information


**Table S1:** fsn371437‐sup‐0001‐TableS1.xlsx.

## Data Availability

The data that support the findings of this study are available from the corresponding author upon reasonable request.
